# Sex dimorphism of cortical water diffusion in normal aging measured by magnetic resonance imaging

**DOI:** 10.3389/fnagi.2013.00071

**Published:** 2013-11-26

**Authors:** Shu-Hang Ng, Wen-Chiun Hsu, Yau-Yau Wai, Jiann-Der Lee, Hsiao-Lung Chan, Yao-Liang Chen, Hon-Chung Fung, Yih-Ru Wu, Ming-Lun Tsai, Jiun-Jie Wang

**Affiliations:** ^1^Department of Medical Imaging and Intervention, Chang Gung Memorial HospitalLinkou, Taiwan, Republic of China; ^2^Department of Medical Imaging and Radiological Sciences, Chang Gung UniversityTaoyuan County, Taiwan, Republic of China; ^3^Department of Neurology, Chang Gung Memorial HospitalLinkou, Taiwan, Republic of China; ^4^Dementia Center, Chang Gung Memorial HospitalLinkou, Taoyuan County, Taiwan, Republic of China; ^5^Department of Electrical Engineering, Chang Gung UniversityTaoyuan County, Taiwan, Republic of China; ^6^Department of Medical Imaging and Intervention, Chang Gung Memorial HospitalKeelung, Taiwan, Republic of China; ^7^Neuroscience Research Center, Chang Gung Memorial HospitalLinkou, Taoyuan County, Taiwan, Republic of China

**Keywords:** mean diffusivity, sex dimorphism, aging, magnetic resonance imaging, diffusion kurtosis

## Abstract

**Background:** The purpose of this study was to examine sex dimorphism in water diffusion in the brain throughout the normal aging process by magnetic resonance imaging.

**Methods:** Diffusion-weighted images covering the majority of the brain were acquired from 77 healthy participants. Both the mean water diffusivity and diffusion kurtosis were calculated from the cortical regions and parcellated according to the template in anatomical automatic labeling. The mean water diffusivity and diffusion kurtosis from both sexes were examined and subsequently correlated with age. Statistical significance was set at a threshold of *p* < 0.01 after correction for multiple comparisons. In regions that reached statistical significance, a linear regression model was performed. Analysis of variance was conducted to determine the interaction between aging and sex.

**Results:** Sex differences were observed for three aspects. First, compared to females, males presented increased mean water diffusivity and a decreased diffusion kurtosis in the frontal and temporal lobes. Second, a widespread age-related increase in mean water diffusivity was observed, which was more significant in the frontal, occipital, and temporal areas and in the cingulum in females. Third, the diffusion kurtosis decreased with aging but only in restricted areas for both sexes. For the interaction of aging and sex, the most significant change was observed with regards to mean diffusivity, mostly in the right amygdala.

**Conclusions:** A sex-related dimorphism in water diffusion throughout the aging process was observed in the cortex using magnetic resonance imaging.

## INTRODUCTION

The human brain evolves through each stage of life as observed using both postmortem histology and *in vivo* imaging. For example, autopsies show a progressive decline in brain weight (Dekaban, 1978). Voxel-based morphometry using magnetic resonance imaging (MRI) has shown a linear reduction in gray matter content with age, especially in males (Good et al., 2001). A reduction of the brain volume in many cortical regions has been observed and has often been attributed to age-related cell death (Scheibel et al., 1975; Meier-Ruge et al., 1978; Devaney and Johnson, 1980; Wong, 2002). In contrast, specific structures, such as the amygdala and thalamus, are relatively preserved, which may suggest a regional variation in the brain’s susceptibility to aging (Tisserand et al., 2004; Grieve et al., 2005; Curiati et al., 2009; Kalpouzos et al., 2009). There is a growing interest in monitoring age-related changes using functional imaging because it is often assumed that functional alterations precede morphological changes. Therefore, changes in brain function, such as those occurring with diffusion and/or perfusion, could be sensitive imaging biomarkers for distinguishing between normal aging and pathological atrophy at the early stages of disease. Furthermore, a better understanding of functional brain evolution may shed new light on neurodegenerative processes.

Water diffusion can be measured non-invasively by MRI using diffusion-weighted imaging (DWI). The directional dependence in water diffusion in the human brain can subsequently be modeled by diffusion tensor imaging (DTI). The mean diffusivity is a derived semi-quantitative index that has been successfully applied in the study of many neurological diseases (Moseley et al., 1990; Basser et al., 1994; Chang et al., 2010; Lo et al., 2010). The mean diffusivity reflects the magnitude of water diffusion. It is expected that the diffusion in the intracellular space is more restricted and smaller in magnitude than that from the extracellular space (Van Zijl et al., 1991). The measured mean diffusivity in biological environments is a balance of the contributions from the two compartments, i.e., intracellular and extracellular. Therefore, the mean diffusivity could potentially provide microstructural information about tissues. Changes in mean diffusivity may be related to pathological conditions where the balance between both compartments is disrupted, such as cell loss in the process of axonal injury (Budde et al., 2009) or degraded integrity of the myelin sheath (Song et al., 2005).

The mean diffusivity in the whole brain has been reported to be nearly stable throughout the majority of adulthood (Chun et al., 2000). However, regional variation occurs. For example, the mean diffusivity in the anterior and central sub-regions of the thalamus is age-related (Ota et al., 2007), as is the mean diffusivity in representative locations in the frontal and occipital white matter (Engelter et al., 2000). In deep gray matter structures, the aging process can have different effects. An increase in both the diffusion anisotropy and mean diffusivity was observed in the caudate nucleus and putamen (Pfefferbaum et al., 2010). Furthermore, the temporal evolution of water diffusion in the human brain may be different between sexes. For example, females have reduced directionally dependent diffusion in the precentral, the cingulate, the anterior temporal white matter, and especially the right deep temporal regions compared to males (Hsu et al., 2008). In contrast, the mean diffusivity was increased in the left frontal lobe of females relative to males (Szeszko et al., 2003). For females younger than 60 years, increased mean diffusivity was found in the right frontal and temporal regions (Naganawa et al., 2003). The reason for a sex-related dimorphism in the mean diffusivity in the brain is not clear. However, evidence of differences in brain structure between the sexes has been reported based on cortical thickness (Sowell et al., 2007) and neuro-morphometry (Lemaitre et al., 2005).

Examination of local and longitudinal changes in water diffusion may be more useful than global measures of diffusivity (Mascalchi et al., 2002), as whole-brain analyses have failed to account for regional variations. However, regional analyses require a predefined region of interest. The selection of such regions is subjective and tedious, and the selected regions are often located in the white matter (Wang et al., 2010). Selection of regions of interest throughout the whole brain is not practical; therefore, an automatic procedure involving image manipulation such as normalization is preferred. However, image manipulation might change the principal direction of the measured diffusion (Alexander et al., 2001). Recently, Lo et al. (2010) proposed a brain parcellation algorithm for calculating water diffusion from the regional cortex throughout the whole brain. With this approach, we can investigate regional diffusion across the whole brain *in vivo* without having to predefine a region of interest; further, this method does not require manipulation of diffusion images.

The hypothesis of our study is that the aging process may change the water balance between the intra- and extracellular spaces, which would subsequently result in observable changes in mean diffusivity as measured by MRI. Based on morphological studies, such changes could be sex-related and region-specific in the cortex. The current study therefore investigated the age-by-sex interaction of water diffusivity in parcellated cortical regions. We investigated the sex-related dimorphism in water diffusion in the brain throughout the normal aging process. DWIs were acquired from healthy Chinese volunteers recruited from the local community. A correlation of water diffusion with age in the parcellated cortical regions was reported for both sexes.

## MATERIALS AND METHODS

This study was approved by the Chang Gung Medical Foundation Institutional Review Board of Linkou, Taiwan, and complied with the Declaration of Helsinki. Each participant gave written informed consent for participation.

### PARTICIPANTS AND CLINICAL WORK-UP

Seventy-seven participants (37 male and 40 female) were recruited from the local community. The mean age of the male participants was 62.8 ± 7.25 years (range of 51–81 years, median age of 61 years). The mean age of the female participants was 60.7 ± 6.79 years (range of 51–78 years, median of 59 years). To calculate the percentage change in diffusion for each decade, the participants were subsequently divided by age into the following three groups: under 60 years (female/male = 22/13), 60–69 years (female/male = 13/16) and at least 70 years (female/male = 5/8). All subjects were screened with a medical history review and physical examination, which indicated that all subjects were free of cognitive impairment.

### IMAGE ACQUISITION

Images were acquired using a 3-Tesla MR scanner (Trio a TIM system, Magnetom, Siemens, Erlangen, Germany). T2-weighted fluid-attenuated inversion-recovery (FLAIR) and three-dimensional (3D) T1-weighted magnetization-prepared rapid acquisition gradient echo (MPRAGE) images were acquired to rule out concomitant neurological disorders.

Diffusion-weighted imagings were acquired using a spin-echo echo planar imaging sequence with the diffusion-weighting gradients applied in three orthogonal directions and using the following parameters: repetition time (TR)/echo time (TE)/flip angle = 3,000 ms/110 ms/90°, field of view = 256 mm^2^, matrix size = 128 × 128 and 20 axial slices with a thickness of 5 mm to cover the majority of the brain. Multiple *b*-values were acquired from 0 to 4,000 s/mm^2^ in steps of 100 s/mm^2^. The single average acquisition time was 7 min, 39 s.

### IMAGE PROCESSING

The image processing was performed in MATLAB R2009b (MathWorks, Natick, MA, USA). The template was created in Statistical Parametric Mapping 8 (Wellcome Department of Cognitive Neurology, University College London, London, UK). The brain parcellation algorithm followed that described by Lo et al. (2010). In short, a customized group template was created from all participants by normalizing each individual’s T1-weighted MPRAGE image to the ICBM152 template followed by averaging. The averaged brain image was smoothed with an isotropic 8 mm Gaussian kernel. To minimize the contamination from cerebrospinal fluid, the maps were filtered by a gray matter mask, which was created by segmenting each individual’s T1-weighted image. Meanwhile, the T1-weighted MPRAGE images from each individual were co-registered to the non-DWIs. The individual co-registered T1-weighted image was then normalized to the study-specific template by affine transformation. The parameter of the inverted affine transformation was then applied to warp the anatomical automatic labeling (AAL) template.

The mean diffusivity was calculated using the diffusion kurtosis imaging model according to Jensen et al. (2005). The average of the diffusion-related index within the regions specified by the AAL template was calculated. Only cortical regions were selected. As a control study, the mean diffusivity was also calculated in the conventional manner between the non-DWI and images with *b*-values of 1,000, 2,000, 3,000, or 4,000 s/mm^2^.

The percent change between sexes was calculated as the difference divided by the average between sexes. The percent change per decade was calculated by Eq. (1), where mean2 and mean1 are the average values of diffusivity in age decade2 and age decade1, respectively

(1) $$\frac{\left(mean2-mean1\right)/mean1}{decade2-decade1}\times 10.$$

### STATISTICAL ANALYSIS

The participants were divided into two groups according to sex. No significant difference in age between groups was noticed. The difference between sexes with regard to both mean diffusivity and diffusion kurtosis was examined by Student’s *t*-test. The correlation with age was tested by Pearson’s correlation. In regions with significance, a linear regression model was subsequently performed. An analysis of variance (ANOVA) was conducted to detect regions with significant interactions between age and sex, in which three age groups were analyzed.

Statistical significance was set at a threshold of *p* < 0.01 (two-tailed) after correction for multiple comparisons in the study.

## RESULTS

**FIGURE 1:**
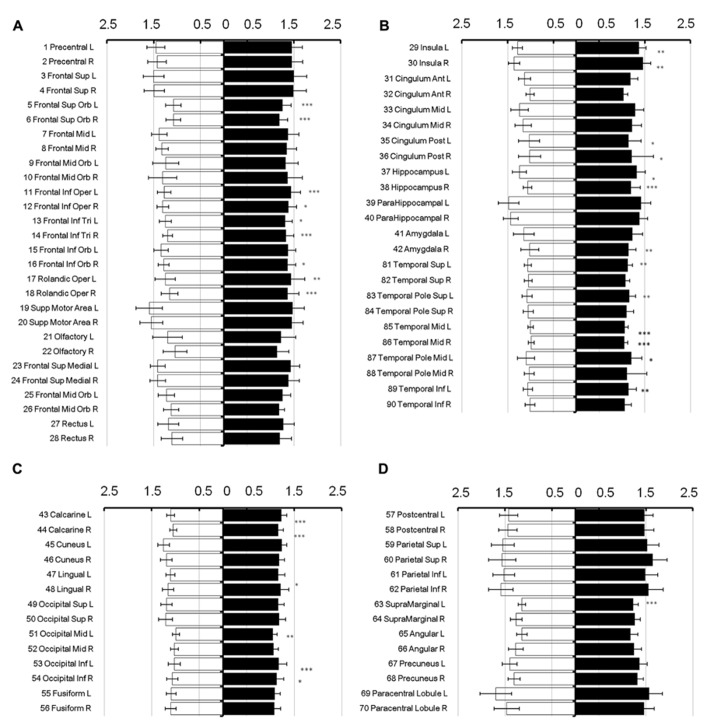
**Mean diffusivity in parcellated cortical regions.** The mean diffusivity in the parcellated cortical regions was plotted for male [filled] and female [blank], respectively. The AAL regions were divided into frontal **(A)**, temporal and cingulum **(B)**, occipital **(C)**, and parietal **(D)**. The mean diffusivity is given in units of 10^-^^3^ mm^2^/s. Asterisk indicates the regions with significance. The regions were numbered according to the anatomical automatic labeling template.

**FIGURE 2:**
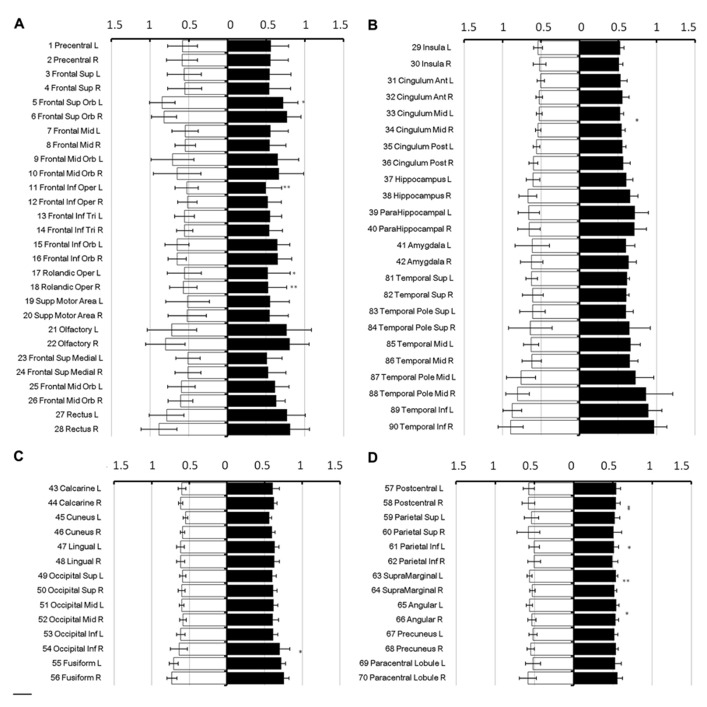
**Diffusion kurtosis in parcellated cortical regions.** The diffusion kurtosis in parcellated cortical regions was plotted for male [filled] and female [blank], respectively. The AAL regions are divided into frontal **(A)**, temporal and cingulum **(B)**, occipital **(C)**, and parietal **(D)**. The regions were numbered according to the anatomical automatic labeling template. Diffusion kurtosis is dimension-free.

To show regional variation, the mean diffusivity in the cortex was plotted in **Figure 1** (male [filled] and female [blank]). For improved visualization, the AAL regions were divided into frontal (**Figure 1A**), temporal and cingulum (**Figure 1B**), occipital (**Figure 1C**), and parietal (**Figure 1D**). Although the water diffusion was relatively stable throughout all of the parcellated brain regions, regional variations were noticeable. The mean diffusivity was approximately (1.23 ± 0.25) × 10^-^^3^ mm^2^/s in the whole brain of females, which was slightly less than that in the male counterparts ([1.28 ± 0.25] × 10^-^^3^ mm^2^/s). The corresponding diffusion kurtosis was plotted in **Figure 2**. The average diffusion kurtosis was similar for both sexes, i.e., approximately 0.61 ± 0.15 in the whole brain.

**FIGURE 3:**
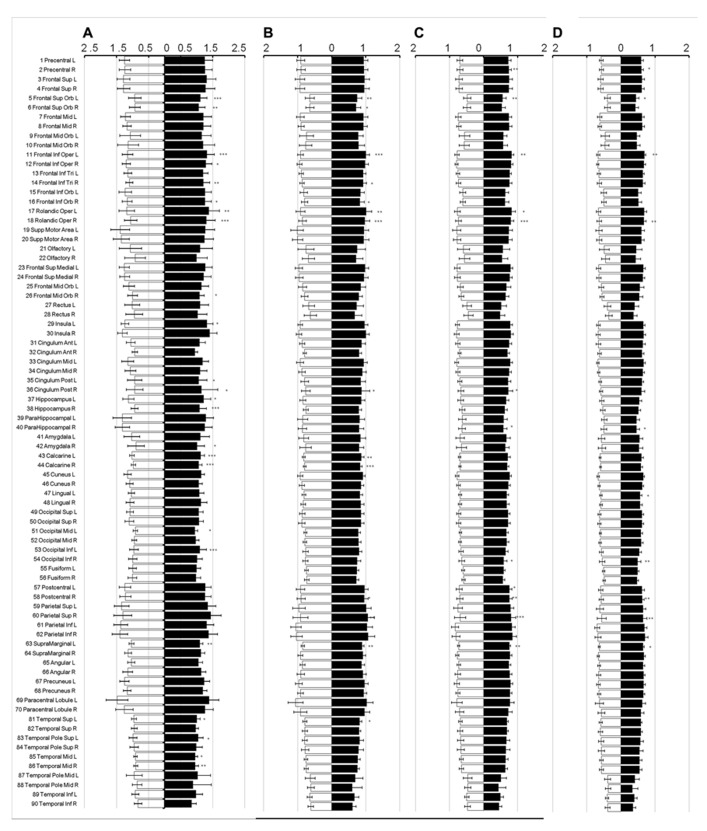
**Mean diffusivity from different diffusion weights.** The figure shows the mean diffusivity calculated between a non-diffusion- weighted image and images with different diffusion weights from 1,000 **(A)**, 2,000 **(B)**, 3,000 **(C)**, and 4,000 **(D)** s/mm^2^. The mean diffusivity within the parcellated cortical regions was plotted for male [filled] and female [blank] and is given in units of *10^-^^3^ mm^2^/s. The regions are numbered according to the anatomical automatic labeling template.

In all brain regions, the mean diffusivity decreased as the diffusion weighting (*b*-value) increased (**Figure 3A**: 1,000 s/mm^2^; **Figure 3B**: 2,000 s/mm^2^; **Figure 3C**: 3,000 s/mm^2^; and **Figure 3D**: 4,000 s/mm^2^). It should be noted that the reduction in the mean diffusivity was not uniform, thus highlighting regional dependence in which the diffusivity decreased more in some regions than in others as the diffusion weighting increased. Furthermore, the mean diffusivity calculated using a diffusion kurtosis model (**Figure 1**) was consistently larger than all of the conventional diffusivities throughout the whole brain (**Figure 3**), even with different *b*-values. Nonetheless, the values calculated using both methods were within a very similar range.

**FIGURE 4:**
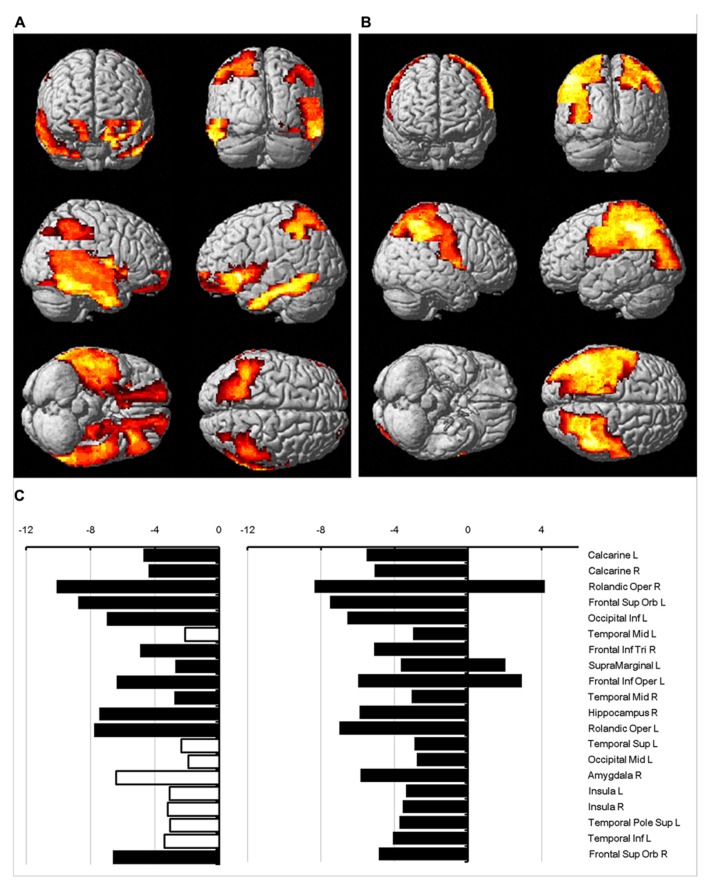
**Percent change in water diffusion.** This figure uses 3D rendering to show regions where the difference in either mean diffusivity **(A)** or diffusion kurtosis **(B)** between sexes is significant. The panel **(C)** plots the percent change between sexes in the mean diffusivity for *b* = 1,000 s/mm^2^ (left), the mean diffusivity (middle), and diffusion kurtosis (right) from regions with significance. The blank bar in the plot of the mean diffusivity calculated with *b* = 1,000 s/mm^2^ indicates that the difference did not reach significance.

The regions where the difference between sexes was significant were rendered in a 3D human brain (**Figure 4A**: mean diffusivity; **Figure 4B**: diffusion kurtosis). **Figure 4C** presents a plot of the percentage changes from the corresponding regions in descending order in terms of *p*-value (from left to right: mean diffusivity using *b* = 1,000 s/mm^2^, mean diffusivity using the diffusion kurtosis model and diffusion kurtosis). The blank bar in the conventional mean diffusivity indicates the percent change in that region, which did not reach significance. Females had reduced mean diffusivity in the calcarine, frontal, occipital, temporal, insular, and cingular regions relative to their male counterparts. In contrast, the differences in diffusion kurtosis were less significant and appeared predominantly as an increase in the female parietal lobe. Changes in conventional mean diffusivity were detected in fewer regions. The regions with changes in mean diffusivity and diffusion kurtosis did not always correspond to each other, which may suggest differing sensitivity to the aging process for both indices.

**FIGURE 5:**
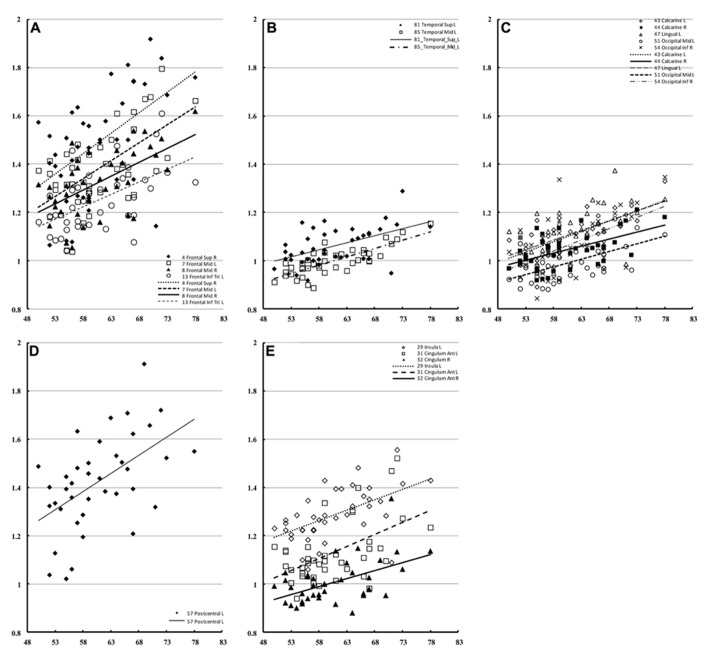
**Regions of interest with significant correlation to female mean diffusivity.** This figure presents a scatter plot of the mean diffusivity against age from selected regions of interest in females where the correlation is significant (*p* < 0.001). The mean diffusivity is given in units of * 10^-^^3^ mm^2^/s, and age is given in years. The regions were divided into the frontal **(A)**, temporal **(B)**, occipital **(C)**, parietal **(D)**, and additional regions **(E)**.

The correlation of water diffusion with age was examined for each sex separately by the Pearson correlation, as summarized in **Table 1**. **Figure 5** plots the mean diffusivity against age in females, which reached significance (*p* < 0.001) in selected regions. A strong correlation between mean diffusivity and age was found in the frontal lobe, including the superior, middle and inferior frontal pars triangularis (**Figure 5A**). The percent increase per decade varied from 9.70% (right middle) to 13.04% (left middle). In the temporal lobe (**Figure 5B**), the increase was noticeable in the left superior (5.96%) and middle (8.23%) regions. In the occipital lobe (**Figure 5C**), the increase was most significant in the right inferior part (9.67%) and lowest in the left lingual gyrus (5.91%). The percent increase in the mean diffusivity per decade in the left postcentral part of the parietal lobe (**Figure 5D**) was 9.57%. Additional regions with significant changes (**Figure 5E**) included the bilateral anterior cingulum (left, 12.65% and right 9.13%) and left insula (5.92%).

**Table 1:** Pearson correlation coefficient from parcellated cortical regions between mean diffusivity and age for each sex.

Regions	Female	Male	Regions	Female	Male
1 Precentral L	0.493**	0.2823	41 Amygdala L	0.1998	-0.0215
2 Precentral R	0.449**	0.3153	42 Amygdala R	-0.1720	0.0538
3 Frontal sup. L	0.484**	0.2474	43 Calcarine L	0.699***	0.418*
4 Frontal SUP. R	0.527***	0.2818	44 Calcarine R	0.547***	0.415*
5 Frontal sup. orb. L	0.114	-0.1017	45 Cuneus L	0.433**	0.1574
6 Frontal sup. orb. R	0.463**	-0.1318	46 Cuneus R	0.492**	0.2895
7 Frontal mid. L	0.602***	0.209	47 Lingual L	0.562***	0.41*
8 Frontal mid. R	0.585***	0.2446	48 Lingual R	0.425**	0.43**
9 Frontal mid. orb. L	-0.1172	0.221	49 Occipital Sup. L	0.3113	0.1538
10 Frontal mid. orb. R	0.0716	0.0434	50 Occipital sup. R	0.358*	0.2689
11 Frontal inf. oper. L	0.35*	0.3148	51 Occipital mid. L	0.585***	0.1946
12 Frontal inf. oper. R	0.412**	0.492**	52 Occipital mid. R	0.459**	0.408*
13 Frontal inf. tri. L	0.548***	0.2559	53 Occipital inf. L	0.334*	0.2228
14 Frontal inf. tri. R	0.434**	0.2128	54 Occipital inf. R	0.524***	0.337*
15 Frontal inf. orb. L	0.2212	0.1027	55 Fusiform L	0.421***	0.2313
16 Frontal inf. orb. R	0.2936	0.1809	56 Fusiform R	0.391*	0.436**
17 Rolandic oper. L	0.446**	0.495**	57 Postcentral L	0.528***	0.372*
18 Rolandic oper. R	0.1857	0.3178	58 Postcentral R	0.413**	0.2888
19 Supp. motor area L	0.37*	0.2664	59 Parietal sup. L	0.36*	0.2292
20 Supp. motor area R	0.363*	0.2492	60 Parietal sup. R	0.3039	0.2472
21 Olfactory L	0.2303	0.0571	61 Parietal inf. L	0.373*	0.2923
22 Olfactory R	0.2053	0.1505	62 Parietal inf. R	0.405**	0.2703
23 Frontal sup. medial L	0.491**	0.2373	63 Supramarginal L	0.2935	0.1111
24 Frontal sup. medial R	0.374*	0.2662	64 Supramarginal R	0.331*	0.436**
25 Frontal mid. orb. L	0.357*	0.2459	65 Angular L	0.2143	0.2177
26 Frontal mid. orb. R	0.2616	0.2259	66 Angular R	0.316*	0.2817
27 Rectus L	0.1874	-0.0897	67 Precuneus L	0.323*	0.2959
28 Rectus R	0.0964	-0.0722	68 Precuneus R	0.373*	0.2706
29 Insula L	0.546***	0.415*	69 Paracentral lobule L	0.1999	0.2683
30 Insula R	0.453**	0.435**	70 Paracentral lobule R	0.348*	0.0867
31 Cingulum ant. L	0.519***	0.1581	81 Temporal sup. L	0.511***	0.459**
32 Cingulum ant. R	0.506***	0.2376	82 Temporal sup. R	0.395*	0.527***
33 Cingulum mid. L	0.3610*	0.1642	83 Temporal pole sup. L	0.39*	0.0046
34 Cingulum mid. R	0.359*	0.0566	84 Temporal pole sup. R	0.2260	0.242
35 Cingulum post. L	0.2364	-0.1932	85 Temporal mid. L	0.79***	0.26
36 Cingulum post. R	0.44**	-0.1391	86 Temporal mid. R	0.434**	0.438**
37 Hippocampus L	-0.0386	0.0503	87 Temporal Pole mid. L	0.0082	0.2782
38 Hippocampus R	0.1267	0.0919	88 Temporal pole mid. R	0.434**	-0.1303
39 Parahippocampal L	0.0933	0.0049	89 Temporal inf. L	0.352*	0.2371
40 Parahippocampal R	0.2778	0.1327	90 Temporal inf. R	0.487**	0.1964

**Table 1** summarizes the Pearson correlation coefficient between the mean diffusivity and age for each sex. *p < 0.05, **p < 0.01, and ***p < 0.001.

**FIGURE 6:**
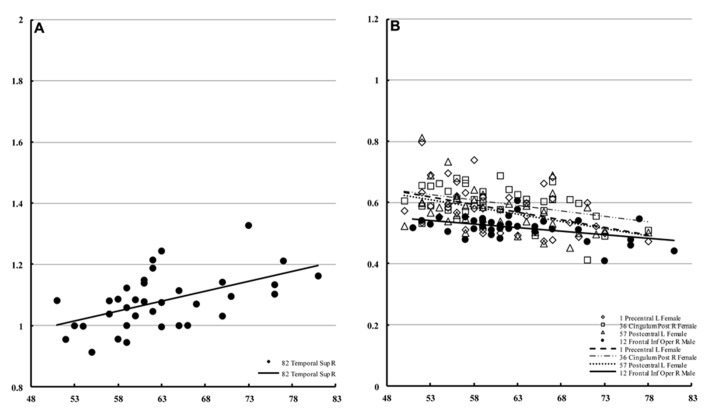
**Regions of interest with significant correlations in male mean diffusivity and in diffusion kurtosis.** This figure is a scatter plot for the age where the correlation is significant (*p* < 0.001) [**(A)** mean diffusivity in males, **(B)** diffusion kurtosis: male, filled; female, blank]. The mean diffusivity is given in units of * 10^-^^3^ mm^2^/s, and age is given in years. The diffusion kurtosis has no dimension.

In males, only the right superior temporal lobe showed a significant correlation with age (**Figure 6A**) with regard to mean diffusivity (percent increase per decade: 7.25%). For diffusion kurtosis, the correlation with age was negative and limited to much smaller regions (**Figure 6B**). In males, the correlation was only observed in the right opercular part of the inferior frontal gyrus (percent decrease per decade: 4.79%), whereas in females the correlation was observed in the left precentral (8.56%), left postcentral (7.71%), and right posterior cingular (9.42%) regions.

**FIGURE 7:**
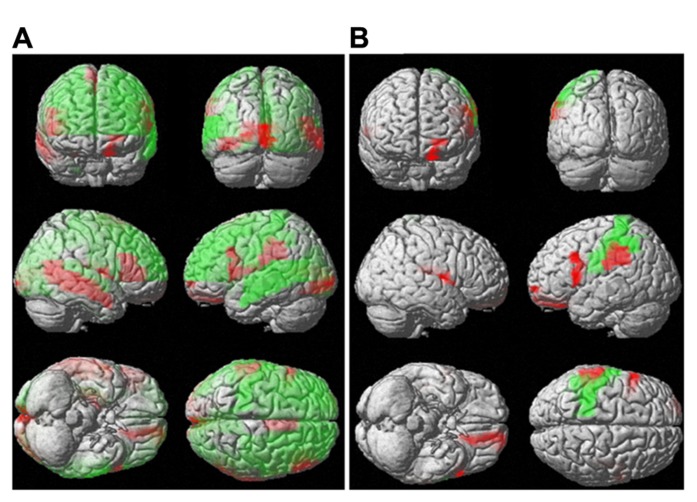
**Interaction between sex and aging for water diffusion.** This figure uses 3D rendering to show the regions where the difference in either the mean diffusivity **(A)** or diffusion kurtosis **(B)** between sexes and aging is significant. The green color indicates significant regions with respect to aging, and the red color shows significance between sexes.

Although the mean diffusivity showed different correlations with aging in both sexes, the interaction between age and sex, as examined by ANOVA, was only significant in the right amygdala (*p* < 0.01). Additional regions that approached significance included the right frontal superior orbital (*p* = 0.037), right posterior cingulum (*p* = 0.016), right hippocampus (*p* = 0.021), and right amygdala (*p* = 0.002). No region showed significance with regard to mean diffusion kurtosis at a threshold of *p* < 0.01. ANOVA analysis showed regions with significant differences in mean kurtosis at a threshold of *p* < 0.05, including the left frontal middle orbital (*p* = 0.026), right frontal inferior opercular (*p* = 0.044), right frontal inferior orbital (*p* = 0.027), right lingual (*p* = 0.041), right temporal superior (*p* = 0.035), and right temporal pole superior (*p* = 0.050) regions. **Figure 7** (**Figure 7A**: mean diffusivity; **Figure 7B**: mean kurtosis) presents a 3D rendering of the human brain where regions with significant differences are color-coded (green: age; red: sex). **Table 2** summarizes the contributions from aging and sex.

**Table 2:** Regions with significance for the sex by age interaction.

Sex	*p*-value		Age	*p*-value	
Region	MD	MK	Region	MD	MK
5 Frontal sup. orb. L	0.000	0.002	1 Precentral L	0.003	NS
11 Frontal inf. oper. L	0.001	0.002	2 Precentral R	0.003	NS
14 Frontal inf tri. R	0.001	NS	3 Frontal sup. L	0.000	NS
17 Rolandic Oper. L	0.007	NS	4 Frontal sup. R	0.000	NS
18 Rolandic Oper. R	NS	0.001	7 Frontal mid. L	0.000	NS
20 Supp motor area R	0.009	NS	8 Frontal mid. R	0.000	NS
38 Hippocampus R	0.000	NS	12 Frontal inf. oper. R	0.001	NS
42 Amygdala R	0.000	NS	13 Frontal inf. tri. L	0.001	NS
43 Calcarine L	0.000	NS	17 Rolandic oper. L	0.001	NS
44 Calcarine R	0.000	NS	19 Supp motor area L	0.009	NS
53 Occipital inf. L	0.001	NS	20 Supp motor area R	0.001	NS
63 Supramarginal L	0.003	0.006	23 Frontal sup. medial L	0.001	NS
86 Temporal mid. R	0.000	NS	24 Frontal sup. medial R	0.001	NS
			29 Insula L	0.002	NS
			30 Insula R	0.003	NS
			31 Cingulum ant. L	0.005	NS
			32 Cingulum ant. R	0.000	NS
			43 Calcarine L	0.000	NS
			44 Calcarine R	0.000	NS
			46 Cuneus R	0.000	NS
			47 Lingual L	0.001	NS
			48 Lingual R	0.000	NS
			50 Occipital sup. R	0.010	NS
			51 Occipital mid. L	0.001	NS
			52 Occipital mid. R	0.003	NS
			54 Occipital inf. R	0.002	NS
			56 Fusiform R	0.005	NS
			57 Postcentral L	0.000	0.002
			58 Postcentral R	0.001	NS
			61 Parietal inf. L	0.001	0.002
			62 Parietal inf. R	0.004	NS
			66 Angular R	0.004	NS
			67 Precuneus L	0.009	NS
			68 Precuneus R	0.004	NS
			81 Temporal sup. L	0.000	NS
			82 Temporal sup. R	0.000	NS
			85 Temporal mid. L	0.000	NS

***Table 2** summarizes the p-values of the contributions from sex and aging, respectively, in parcellated cortical regions where the two-way analysis of variance reached significance for mean diffusivity (MD) and diffusion kurtosis (MK). NS, non-significance.*

## DISCUSSION

The main finding of the current study is that functional alterations of the human brain, as reflected by water diffusion, are sex-dependent and region-specific and evolve with aging. The novelty of this study is that this is the first use of an automatic procedure to measure the mean diffusivity and diffusion kurtosis of water in parcellated cortical regions. The current study reports that differences in diffusion properties can occur between sexes in widespread cortical regions. In many brain regions, the mean diffusivity is larger and the diffusion kurtosis is smaller in males. More severe and widespread changes were observed in males than in females.

Furthermore, the correlative analysis indicates that males and females age at different rates. An age-related increase in mean diffusivity was observed in females, but the increase was less significant than that in their male counterparts. The mean diffusivity and mean kurtosis may have different sensitivity and specificity for characterizing aging, and each provides different types of information regarding the underlying tissue environment. Diffusion kurtosis could decrease with aging, but in fewer areas than decreases in mean diffusivity. These observations suggest a microstructural alteration in the normal aging process that can be expressed as a loss of tissue complexity, cell loss, and subsequent alteration of the water balance between compartments.

Finally, regarding the interaction of aging and sex, the ANOVA analysis indicated that the most significant change occurred in the right amygdala, according to the mean diffusivity. Consistent with our hypothesis, sex-related dimorphism throughout the aging process was observed.

### SEX-DEPENDENT DIMORPHISM OF DIFFUSION

The interaction between sex and age with regard to diffusion is a complicated phenomenon that could involve regions, properties (mean diffusivity and diffusion kurtosis), sex, aging, and combinations of the above effects. Sex-related differences in water diffusion could occur in many brain regions. For example, there was an increase in mean diffusivity in the brain in males relative to females. In contrast, the mean diffusivity increased with age in females but not in males.

Increased mean diffusivity in the white matter is more noticeable in males than in females, and such white matter includes the right superior longitudinal fasciculus and the right inferior longitudinal fasciculus (O’Dwyer et al., 2012). A reduction in diffusion kurtosis was observed in the prefrontal regions in an aging study and was attributed to a loss of structural complexity (Falangola et al., 2008). In contrast, the current study reports a change in water diffusion in various parcellated brain areas rather than a few selected regions of interest or white matter alone.

Age-related sex differences in atrophy have been reported in the middle part of the right temporal lobe, the left basal ganglia, the parietal lobe, and the cerebellum in male subjects but not in female subjects (Xu et al., 2000). The affected cortical regions in our study are largely consistent with those identified in volumetric studies, and the effect may be more severe in males than females. The diffusion changes in our study are consistent with observations that brain atrophy is age-related, sexually dimorphic, lateralized, and region-specific, in addition to being more prevalent in men than in women (Cowell et al., 1994).

Most studies on diseases using DTI, including those evaluating Alzheimer’s disease and mild cognitive impairment, have assumed that functional changes are secondary to gray matter atrophy (Bosch et al., 2012). These studies have predominantly focused on white matter because of the nature of the diffusion tensor model (Wang et al., 2010). Our hypothesis is that diffusion changes in the cortical gray matter may precede morphological loss, and in the present study, we observed increases in mean diffusivity in many cortical regions. Increased mean diffusivity is often attributed to increased extracellular space, which is subsequently filled by cerebrospinal fluid that has a larger mean diffusivity, and increased mean diffusivity is often attributed to cell death in the underlying microstructure. Therefore, the increased water diffusivity and decreased diffusion kurtosis in our study could be a result of increased extracellular space, which may be related to a loss in micro-environmental complexity that most likely results from cell death.

The reason for the observed sex difference is still under investigation. However, sex-related differences were observed for synaptic protein loss in various parts of the cortex in a study on synaptophysin- and synaptosomal-associated protein (Downes et al., 2008). The hormone difference between sexes may have a neuro-protective effect (Turgeon et al., 2006). The increased mean diffusivity in males may reflect an elevated level of microstructure damage. However, no significant difference in cognitive impairment was found between sexes, which suggests that a difference in the brain reserve may exist between sexes.

### RELATION OF AGING WITH SEX-DEPENDENT DIMORPHISM OF WATER DIFFUSION IN THE BRAIN

The aging process affects different brain regions in the different sexes. In general, mean diffusivity in males, although elevated relative to that in females, is relatively stable with aging. In the female brain, the mean diffusivity in many regions increased with age. The increase in cortical diffusion in females during the aging process is most prominent in the frontal lobe and anterior cingulum. Such regional dependence of the aging effect can be attributed to different factors, such as lasting morphological changes and/or actual changes in the mean diffusivity (Camara et al., 2007). Furutani et al. (2005) reported an age-related increase in the mean diffusivity in the gray matter, especially in the lentiform region. In contrast, several studies have reported that a lack of age-by-sex interaction in the white matter (Hsu et al., 2008; Wu et al., 2011; O’Dwyer et al., 2012). The discrepancy between previously observed atrophic decay effects and the findings of our study may be related to the different imaging mechanisms used and variations in detection sensitivity in functional approaches, such as in diffusion imaging. The biological basis of this process is still largely unclear but may be attributed to differences in the degenerative process, although this notion requires further validation.

Diffusion kurtosis imaging has emerged as a new imaging technique with applications in many neurological diseases such as in the analysis of tumors (Raab et al., 2010), epilepsy (Akiyama et al., 2012), and Parkinson’s disease (Wang et al., 2011). The observed changes in diffusion kurtosis and mean diffusivity are largely related to a deviation from the Gaussian distribution in water molecules. Because such a deviation in the biological environment results from restriction of diffusion, the change in the diffusion kurtosis may be related to the evolution of structural complexity. Reduced diffusion kurtosis, together with increased mean diffusivity, may suggest increased extracellular space within the voxel of interest. An age-related, non-Gaussian diffusion process was previously reported in the prefrontal brain, and this diffusion decreased after the age of 47 years (Falangola et al., 2008). The current study reports that the correlation of diffusion kurtosis with aging is region-specific and more significant in females.

### INTERACTION BETWEEN SEX AND AGING FOR DIFFUSION

The most significant interaction between sex and age from the ANOVA analysis involved mean diffusivity in the right amygdala. The interaction appeared to display a hemispheric effect. Age-related differences in the functional connectivity of this region with the rest of the brain have been previously reported (St Jacques et al., 2010). Differences in the amygdala have been noticed between sexes in terms of size (Goldstein et al., 2001), lateralization (Cahill et al., 2001), cognitive function (Ziabreva et al., 2003; Hamann, 2005; Lanteaume et al., 2007), and the presence of excitatory neurons (Cooke and Woolley, 2005). The involvement of the amygdala determined by our analysis supports an increased sensitivity of functional imaging over morphological measurements. Many reports have shown that the aging process also involves the amygdala, as determined by activation, volumetric, or functional connectivity data (St Jacques et al., 2010; Todd et al., 2011; Shen et al., 2013). Given the role of the amygdala in emotional processing (Adolphs et al., 1994; Morris et al., 1998) and memory modulation (Hamann et al., 1999; McGaugh, 2004), future studies on aging-related cognitive changes may need to include the function of the amygdala as a covariate.

### INTERPRETATION OF CHANGES IN DIFFUSION

In this study, mean diffusivity was calculated from two different models: the non-Gaussian model using diffusion kurtosis imaging and, for comparison, the conventional Stejskal–Tanner model (Stejskal, 1965). The conventional model assumes free diffusion and is currently used in routine clinical practice. The mean diffusivity calculated from conventional model is generally lower, as observed in selected brain regions in rats (Veraart et al., 2011). The current study confirms this finding in humans and furthermore indicates that the reduced diffusivity is a general phenomenon in all brain regions. The difference between the two models is most likely related to the fact that the diffusion-related signal decay was fit to a parabola in the non-Gaussian model (Jensen and Helpern, 2010) rather than to a straight line as in the conventional diffusion model.

Although the mean diffusivity in the diffusion kurtosis model is different from that in the conventional model, the diffusion kurtosis model did not detect a larger difference between sexes. However, the mean diffusivity did identify more regions with significant differences, most likely because of reduced variation in the measurements (**Figure 3**), as the mean diffusivity in the study was calculated from multiple acquisitions with different diffusion weights, which may improve the signal-to-noise ratio.

Furthermore, a decrease in mean diffusivity with an increase in diffusion weighting was noticed throughout the brain. The *b*-value dependence of mean diffusivity has been reported in various brain regions (DeLano et al., 2000; Hui et al., 2010; Veraart et al., 2011). We found that such dependence may occur in all regions within the whole brain. Additionally, as the diffusion weighting increased, the *b*-value dependence decreased and reached saturation. The *b*-value dependence is thus attributed to the selective suppression of the diffusion component. The mean diffusivity calculated from low-diffusion-weighting acquisitions demonstrated a higher difference between sexes. This phenomenon should be further investigated in the future.

In summary, because the calculation of mean diffusivity can be affected by diffusion weighting and by imaging parameters, particular caution should be exercised in studies using data from multiple centers. In addition, different models have different assumptions and, as a result, different sensitivities. The calculation of mean diffusivity from the diffusion kurtosis imaging model is a good choice because of its increased sensitivity and reduced variation. Future studies should evaluate the effect of the adopted diffusion model on the measurement.

### STUDY LIMITATIONS

The current study used AAL as a template for normalization. However, because AAL is based on Caucasian brains, which differ in many aspects from Asian brains, potential bias may have been introduced in measurement of deep gray matter and nuclei. A gray matter mask was produced to reduce contamination from cerebrospinal fluid and white matter. The current study only focused on gross anatomical structures such as the lobes of the brain. Further information for these nuclei may be obtained by improved image co-registration procedures, which may lead to a new understanding of the role of nuclei in sex-related differences in aging.

Because there is currently no well-established model for diffusion anisotropy in gray matter, our study only focused on investigating mean diffusivity and diffusion kurtosis. However, in future investigations, both the diffusion tensor and diffusion kurtosis tensor will be acquired in an effort to develop a more comprehensive interpretation of the observed sex dimorphism.

## CONCLUSION

Magnetic resonance imaging was used to observe sex dimorphism in the diffusion of water in the cortex, which was found to be region-specific and to evolve differently during the normal aging process.

## Conflict of Interest Statement

The authors declare that the research was conducted in the absence of any commercial or financial relationships that could be construed as a potential conflict of interest.

## Author Contributions

Shu-Hang Ng and Wen-Chiun Hsu have contributed equally to this work. Guarantor of the integrity of entire study: Jiun-Jie Wang. Study concepts/study design: Jiun-Jie Wang and Shu-Hang Ng. Data acquisition or data analysis/interpretation: Yau-Yau Wai, Wen-Chiun Hsu, Jiann-Der Lee, Yao-Liang Chen, and Hsiao-Lung Chan. Manuscript drafting or manuscript revision for important intellectual content: Shu-Hang Ng, Yao-Liang Chen, Hon-Chung Fung, Yih-Ru Wu, and Jiun-Jie Wang. Manuscript final version approval: all authors. Literature research: Wen-Chiun Hsu and Yu-Chun Lin, Ming-Lun Tsai. Clinical studies: Wen-Chiun Hsu, Hon-Chung Fung, and Yih-Ru Wu. Statistical analysis: Ming-Lun Tsai and Yau-Yau Wai. Manuscript editing: Jiun-Jie Wang and Shu-Hang Ng.
